# Early Diagnosis of Systemic Sclerosis: The Role of General Practitioner

**DOI:** 10.7759/cureus.32291

**Published:** 2022-12-07

**Authors:** Armanda Freixo, Cecília Abreu

**Affiliations:** 1 Unidade de Saúde Familiar (USF) Lethes, Unidade Local de Saúde do Alto Minho, Ponte de Lima, PRT

**Keywords:** red flags, systemic sclerosis, classification, early diagnosis, very early systemic sclerosis

## Abstract

Systemic sclerosis (SSc) is a chronic, rare, and idiopathic disease characterized by the presence of microcirculatory and immune alterations followed by fibrosis. It is clinically heterogeneous and may present a rapid and progressive involvement that leads to disability and death. Over the years, the approach has changed with an increasing focus on early diagnosis.

Raynaud's phenomenon (RP) and puffy fingers are "red flags" to refer the patients to rheumatology to detect and start the appropriate treatment of such a rare and complex disease.

We present a case of a 75-year-old woman with edema and bright erythema on the back and palm of the hands, telangiectasias of the face, and RP with three years of evolution.

The aim of this case is to recall the importance of primary care physicians in recognizing the main clinical manifestations of SSc that are sometimes undervalued.

## Introduction

Systemic sclerosis (SSc)/scleroderma is a rare and idiopathic connective tissue disease (CTD) [1,2] characterized by the presence of microcirculation (with tissue hypoxia), immune alterations (changes in lymphocyte function and production of autoantibodies), and fibrosis (excessive production of collagen with fibrosis) [3]. It is mostly seen in women and has a peak incidence between the third and fifth decade of life [4].

SSc is the CTD with the highest mortality [3]. William Osler defined it as “one of the most terrible of all human diseases,” causing functional limitation and diminishing people's quality of life [5].

Patients with SSc are divided into two subgroups: limited cutaneous systemic sclerosis (lcSSc) and diffuse cutaneous systemic sclerosis (dcSSc) [6]. The main difference is in disease progression and the extent of cutaneous and visceral involvement. In lcSSc, which is the most common subgroup [1,2], sclerosis is limited to the face and extremities and has a more insidious onset. In contrast, dcSSc causes greater morbidity and mortality, has faster progression, and greater sclerosis and visceral involvement.

Although rare, it is a clinically heterogeneous disease, not expressing itself in the same way in all patients, ranging from mild clinical manifestations to a multiplicity of systemic conditions, involving internal organs, which can cause devastating consequences, including death [7]. Examples of this are scleroderma renal crisis, sudden cardiac death, interstitial lung disease, pulmonary hypertension, and digital ulceration [8].

Due to the complexity of this disease, its approach has changed over the years to facilitate SSc diagnosis at the earliest stage and to start the appropriate treatment when microvascular remodeling, tissue fibrosis, and atrophy are not already irreversible [9].

For decades, the existence of an early stage of SSc has been recognized, without skin involvement, which can progress to a definitive disease [10].

It is currently consensual that the existence of Raynaud's phenomenon (RP) and the presence of puffy hands are "red flags" that alert a physician to suspect very early SSc and, therefore, in these situations, the patient should be referred to a rheumatologist for eventual final diagnosis and to carry out a close follow-up to timely detect the beginning of internal organ involvement and start an appropriate therapy [7].

## Case presentation

We present a case of a 75-year-old female patient, who was independent in daily life activities. She was retired and had a low socioeconomic level. She previously worked in a food store and had moderate alcohol consumption. As personal antecedents, she had high blood pressure (controlled) and chronic obstructive pulmonary disease (COPD).

The patient attended the scheduled appointment for arterial hypertension in the context of primary health care, in which she reported usual dyspnea for medium exertion. No other complaints were reported.

**Figure 1:**
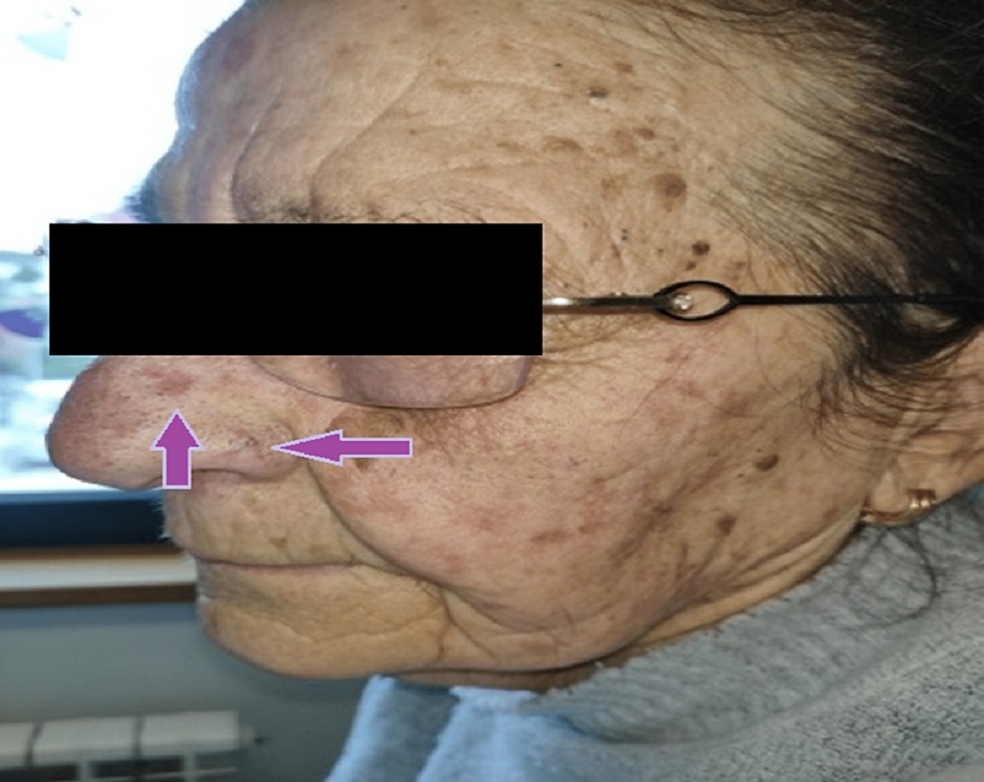
Telangiectasias of the face The arrows show widened threadlike vessels.

**Figure 2:**
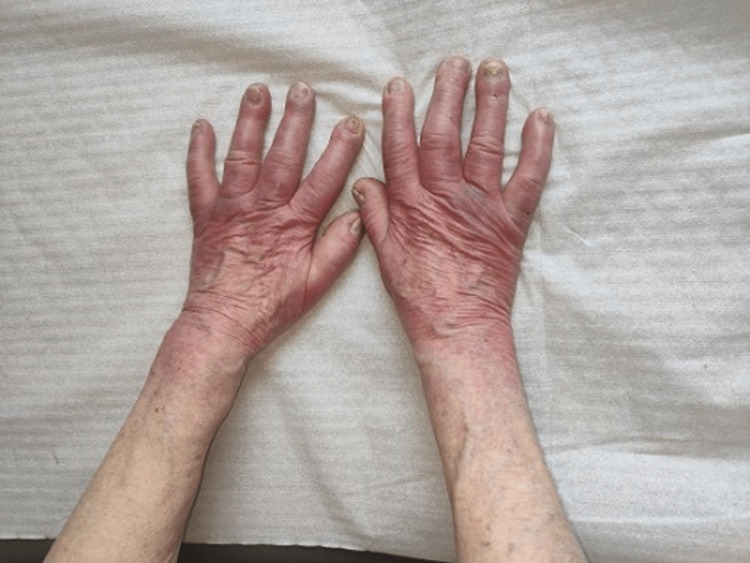
Puffy hands/puffy fingers

On physical examination and without clinical relevance for the patient, the presence of edema and bright erythema on the back and palm of the hands was observed (glove shape), with scars of "chilblains" on the tips of the fingers, which the patient referred to be frequent, as well as telangiectasias of the face (Figures 1, 2).

When questioned, she mentioned episodes of paleness, followed by blue discoloration and redness of the hands bilaterally, associated with diffuse edema and erythema on the dorsal aspect of the hands, without associated pain or numbness, especially in winter and with three years of evolution.

Still, in the clinical history, the patient referred fatigue for medium efforts. She denied skin thickening, dysphonia, dysphagia, or reflux. No intestinal changes, arthralgia, swelling of the joints, photosensitivity or malar rash, oral or genital ulcers, and no dry complaints were reported.

According to the medical records prior to this observation, the patient presented an episode of worsening usual dyspnea and cough three years ago, which was considered an exacerbation of COPD and was treated with oral corticosteroids. No further complications to date were noted.

Based on these physical findings and clinical history (RP + puffy hands), it was decided to refer the patient to rheumatology to continue the evaluation and definitive diagnosis.

The patient was later observed by rheumatology. The findings corroborate the clinical history and the findings in the objective examination, posing the hypothesis of “probable very early diagnosis of systemic sclerosis (RP + puffy hands + telangiectasias).” Following this observation, capillaroscopy, analytical study with immunological profiles, including antinuclear antibodies (ANA), topoisomerase I antibodies (Scl-70), anticentromere antibodies, and antibodies to ribonucleoprotein (RNP), organ study with respiratory function tests, echocardiogram, and high-resolution computed tomography (HRCT) of the chest were scheduled.

The patient started nifedipine 15 mg/day with monitoring of the blood pressure profile and reassessment in five weeks with an improvement of the symptoms. Six months later, she maintains follow-up at the consultation with strict follow-up and complementary exams requested despite not having active CTD.

## Discussion

The aim of treatment in SSc is to improve the quality of life of patients, minimizing organic damage and, consequently, the risk of life. To date, many developments have been made to improve the understanding of mechanisms involved in the pathogenesis of the disease. Despite that, the diagnosis of SSc still remains very difficult, especially in the early stage, when patients are oligosymptomatic [8].

Very early SSc is defined by the presence of RP, puffy fingers (PF), disease-specific autoantibodies, and microvascular alterations in capillaroscopy (requiring at least two, or better, all three items to be present) [11].

RP and puffy hands/fingers (PF) (digital edema) are the earliest signs to suspect the presence of SSc [12,13]. Another review study wrote about the final analysis conducted by the European League Against Rheumatism Scleroderma Trials members, which considered RP and puffy swollen hands warning signs for the general practitioner/family doctor to suspect very early SSc and thus, to refer the patient to a specialist for the final diagnosis [13].

However, these early disease features are not specific to SSc and are also present in other CTDs, such as undifferentiated connective tissue disease (UCTD) and mixed connective tissue disease (MCTD). Because of this, it is important to underline that the presence of RP should always be a reason for a more detailed investigation. Nowadays, it is clear that for a patient with RP, SSc should be ruled out by the general practitioner by referral to a rheumatologist to carry on more specific exams [8].

With regard to very early SSc, the decision between treating and not treating remains a dilemma, due to the risk of overtreatment and iatrogenesis [5]. There is no agreement on the predictors capable to identify patients who will develop an established disease. So, for the moment, the only viable clinical strategy remains close clinical follow-up [14].

## Conclusions

The aim of this clinical case is to recall the earliest clinical manifestations of SSc, allowing an early diagnosis and a greater celerity in the guidance before a probable case of SSc.

The contribution of the family doctor is likewise highlighted in this case, by making this early recognition and in the communication between the different levels of care and management of the various medical specialties that each patient needs.
